# Effectiveness and safety of intracardiac electrocardiogram guidance for epicutaneo-cava catheters via the lower extremity in preterm infants: a retrospective study

**DOI:** 10.1186/s12887-023-04444-w

**Published:** 2023-12-09

**Authors:** Xinying Yu, Li Gai, Xuejun Wang, Chaonan Kong, Na Cao, Ling Fan, Fan Yang, Xiaoyu Yang, Le Sun

**Affiliations:** 1 Department of Pediatrics, Shengjing Hospital of China Medical University, Shenyang City, 110004 Liaoning Province People’s Republic of China; 2 Department of Nursing, Shengjing Hospital of China Medical University, Shenyang, China; 3 School of Nursing, China Medical University, Shenyang, China

**Keywords:** Epicutaneo-Cava catheters, Intracardiac electrocardiogram, Lower extremity, Nursing, Preterm infant

## Abstract

**Introduction:**

In recent years, intracardiac electrocardiogram (IC-ECG) technology has been widely used for epicutaneo-cava catheter (ECC) placement and has shown many potential advantages. However, evidence about the quantitative changes, effectiveness, and safety of IC-ECG for lower extremity ECC is sparse. This study aimed to explore the quantitative changes in IC-ECG for lower extremity ECC and determine its effectiveness and safety.

**Methods:**

A retrospective study was conducted on 303 premature infants who underwent successful IC-ECG-guided lower extremity ECC placement between January 2019 and December 2021. All patients underwent chest X-ray postoperatively to verify the position of the catheter tip. The amplitudes of the surface electrocardiogram and IC-ECG QRS waves and the difference between the two amplitudes were measured. The effectiveness (matching rate between IC-ECG and chest X-ray) and safety (incidence of catheter-related complications) of IC-ECG for lower extremity ECC were evaluated.

**Results:**

The matching rate between IC-ECG and chest X-ray was 95.0%. When the catheter tip was optimally positioned, the QRS amplitude of the IC-ECG was 0.85 ± 0.56 mv higher than that of the surface electrocardiogram. The overall incidence of catheter-related complications was 10.6%. The actual ECC insertion length was associated with a noticeably increased risk of catheter-related complications.

**Conclusions:**

This study suggests that IC-ECG is an effective and safe method by observing the dynamic changes in both QRS complexes and P wave to locate the tip of lower extremity ECC in preterm infants. Our findings would facilitate the application of IC-ECG for ECC localization.

## Introudction

Epicutaneo-cava catheter (ECC) procedures have been widely used in clinical practice. Catheter tip location plays a crucial role in promoting catheter effectiveness, as a central location has significantly lower complication rates, while malposition may cause catheter malfunction and future complications [1–3]. Consequently, tip location has always been a hot topic in ECC research. The latest guideline recommends using methods to identify the tip location of central venous catheters during the insertion procedure [4]. Real-time ultrasound and intracardiac electrocardiogram (IC-ECG) are the most commonly used methods. However, ultrasound examination requires specialized skills, and bedside ultrasound devices are not as common as bedside ECG monitors. Thus, the use of IC-ECG has been increasing, and several studies have confirmed its feasibility, effectiveness, accuracy, and safety [3, 5–18]. The latest meta-analysis shows that IC-ECG can improve the accuracy of ECC tip localization and reduce the incidence of related complications [19].

IC-ECG is a non-visual method for catheter tip location [15]. It is based on dynamic morphology changes of the P wave when the catheter tip is located in the superior vena cava (SVC). In brief, the P wave becomes higher when the catheter proceeds into the SVC and reaches its peak at the cavo-atrial junction. Deeper on, a significant reduction in the P wave or a biphasic P wave indicates that the catheter has entered the right atrium. In recent years, IC-ECG has been widely used for ECC placement in neonates [6, 8, 10, 11, 14, 16–18, 20]. Because the majority of studies have suggested that the upper extremity was the preferred choice for neonatal catheterization, most studies on IC-ECG focused only on upper extremity ECC with the catheter tip in the SVC and excluded lower extremity ECC with the catheter tip in the inferior vena cava (IVC).

However, growing evidence shows that lower extremity ECC may have more significant advantages than upper extremity ECC in neonates [11, 21, 22]. The 2016 guidelines of the American Infusion Nurses Society (INS) clearly state that lower limb catheterization is appropriate in newborns [23]. Chinese clinical practice guidelines also recommend that the lower extremity is preferred for neonatal catheterization [24]. Based on previous research and guideline recommendations, in recent years, an increasing number of lower extremity ECCs have been applied to newborns.

Nevertheless, evidence about the application of IC-ECG for lower extremity ECC is sparse. Only three studies reported the IC-ECG changes with the catheter tip located in the IVC [11, 18, 20]. When the tip is in the IVC, the QRS amplitude increases continuously as the catheter approaches the heart. As the catheter enters the right atrium, the P wave becomes upright, and the amplitude dramatically increases. At this point, the ECC needs to be withdrawn until the P wave returns to normal size. These three reports only described this phenomenon qualitatively without data to support it. Hence, the quantitative changes of IC-ECG for lower extremity ECC remain unknown. Based on previous clinical practice, we have accumulated some valuable IC-ECG data on lower extremity ECCs. In order to understand the main changes, effectiveness and safety of IC-ECG in lower extremity ECC, we conducted this preliminary retrospective study to provide support for future experimental studies. To the best of our knowledge, this is the first study to focus on quantitative changes in IC-ECG for lower extremity ECC.

## Methods

### Study design and population

**Fig. 1:**
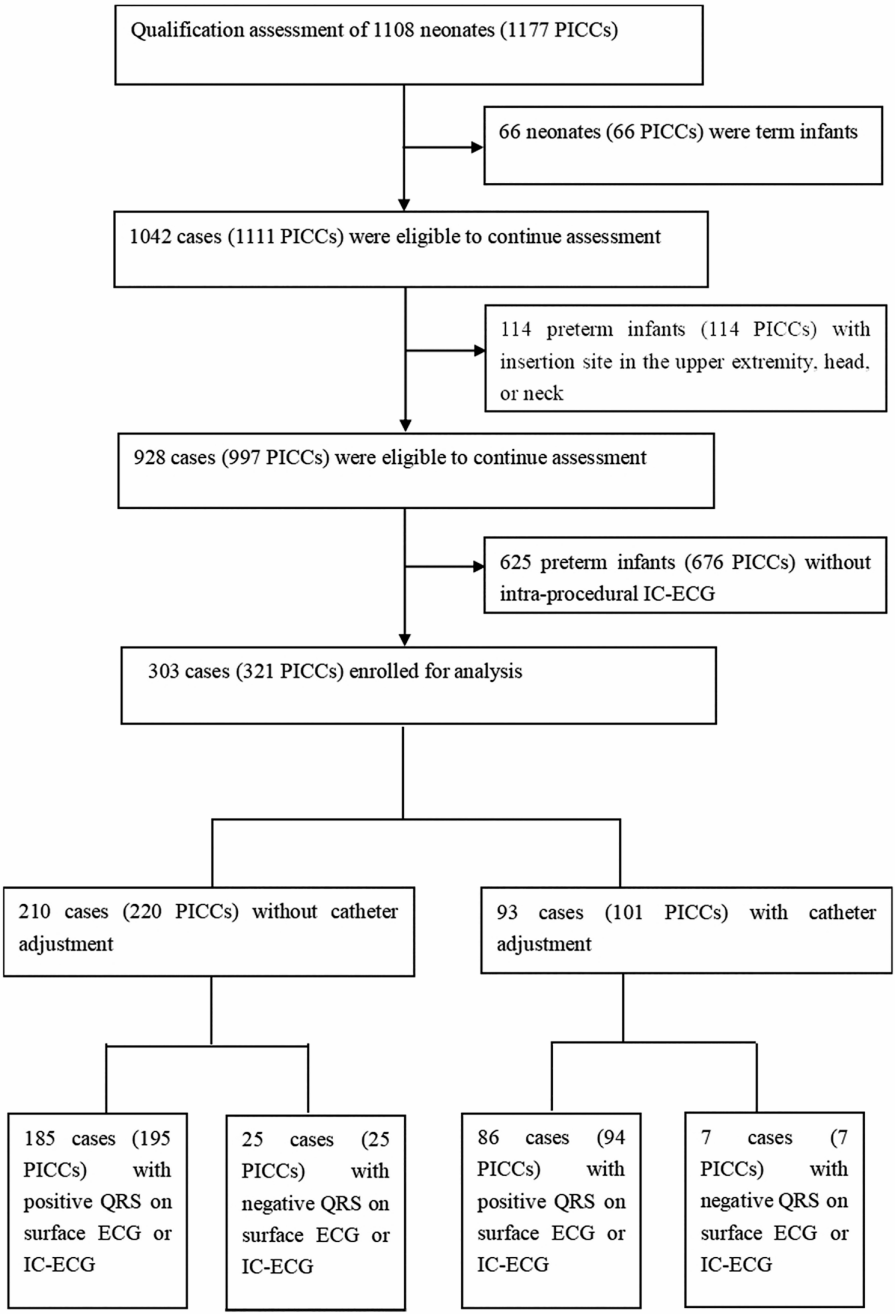
CONSORT flow diagram

This study was a retrospective observational study, guided by the STROBE tool. This study was conducted in a tertiary-level neonatal intensive care unit (114 beds). The subjects were hospitalized preterm infants (gestational age < 37 weeks) between January 2019 and December 2021 who underwent IC-ECG-guided lower extremity ECC placement. Exclusion criteria were as follows: ambiguous chest X-ray(CXR), unexplained IC-ECG, or incomplete ECG (complete ECG should include surface ECG and IC-ECG). Finally, 303 preterm infants (321 ECCs) were included in the study (Fig. 1).

### IC-ECG guidance for ECC placement

**Fig. 2:**
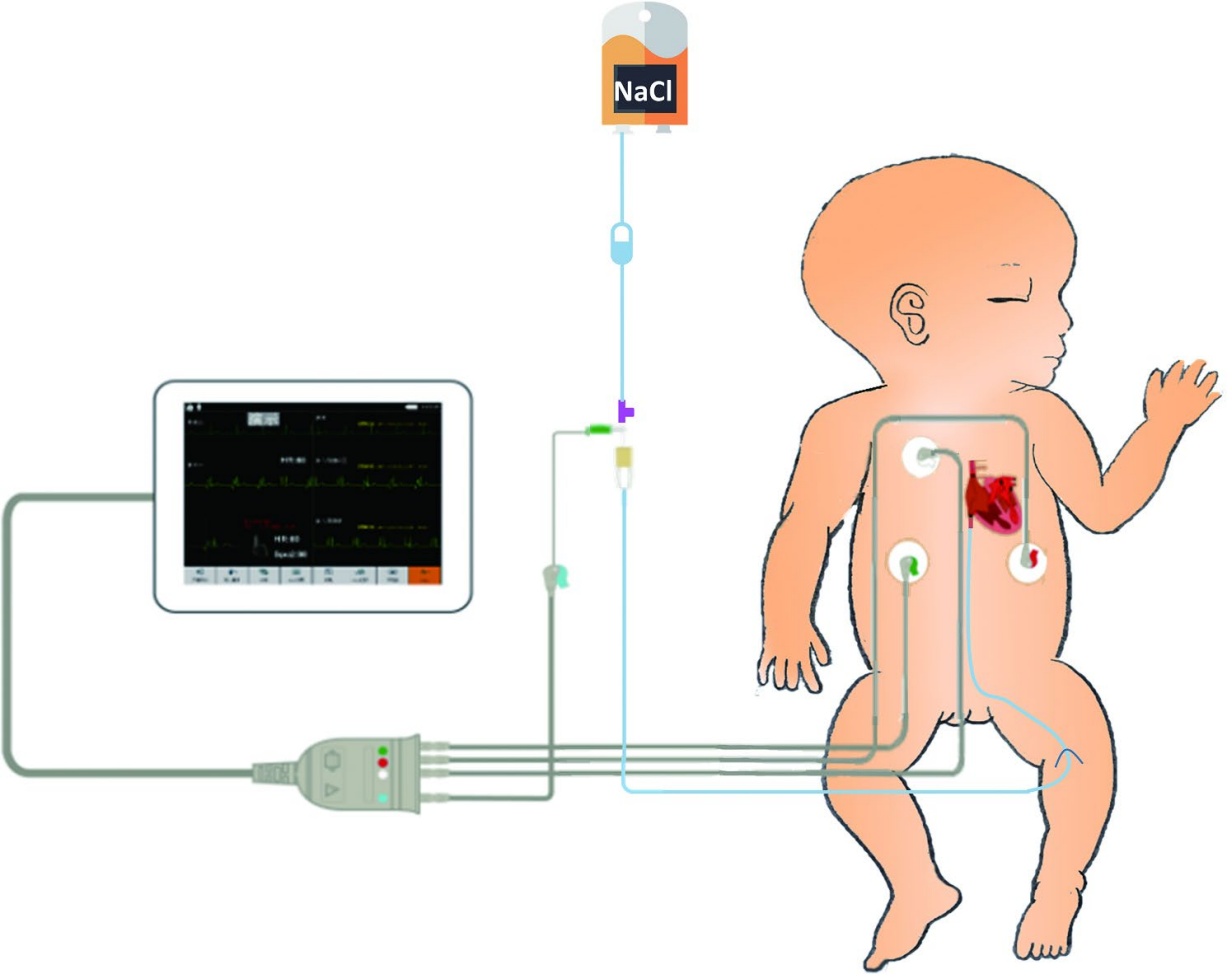
Connection of the electrodes for IC-ECG.

In our study, all catheters used were 1.9 Fr with a single lumen (UNI-ECC, Haolang Technology [Foshan] Co., Ltd). The ECG instrument was an ECG monitor (COMEN C100B, Shenzhen Comen Medical Instruments Co., Ltd) with four electrodes placed on the left subclavian, right subclavian, left upper abdomen, and right upper abdomen. Before insertion, preterm infants had their surface ECG assessed, and a reference length was measured. The reference length was equal to the length of “the insertion site - the middle of the groin - navel - xiphoid” [25]. When the catheter is inserted near the reference length and blood is withdrawn smoothly, the upper left electrode is connected to the ECC with a heparin cap, scalp needle, and electrocardiogram electrode lead clip. A column of saline in the catheter is used as an intracavitary electrode. The connection of the four electrodes for IC-ECG is shown in Fig. 2. All catheters were inserted blindly through a lower limb vein (femoral or great saphenous vein) by certified ECC nurses.

Preterm infants were kept in a supine position and remained comfortable and quiet. The main procedures included sterilization, puncture, catheterization, application of IC-ECG, fixation, and post-procedural CXR [18]. ECC insertion was guided by observing sequential and characteristic alterations in QRS complexes and P waves. The QRS amplitude increased continuously as the catheter deepens. Deeper on, an upright P wave was present, at this point the catheter was withdrawn until the P wave returned to normal, and the catheter was fixed at the inferior cavo-atrial junction where the QRS amplitude increased and the P wave remained normal [11, 18, 20].

### Outcome indicators

The outcomes included the effectiveness, quantitative changes, and safety of IC-ECG for locating the tip of lower extremity ECC in preterm infants.

Effectiveness was assessed based on the matching rate between IC-ECG and CXR. In this study, matching was defined as an increased QRS amplitude in intraoperative IC-ECG and confirmation of the catheter in the IVC via postoperative CXR. A tip located at the junction between the IVC and right atrium (above the level of the diaphragm within the IVC) was defined as the optimal central location [4, 26].

Subjects were divided into two groups by whether the catheter was in the optimal central location, the quantitative changes were obtained by measuring the amplitude of IC-ECG QRS wave (IR), the amplitude of the surface ECG QRS wave (SR), and the difference between IR and SR (IR-SR) between the two groups.

Safety was assessed based on whether catheter-related complications occurred. Catheter-related complications included extremity swelling, phlebitis, catheter-related blood stream infection (CRBSI), and leakage. The nurses evaluated catheter function and observed catheter-related complications daily. After ECC insertion, if there was visible thickening of the extremity at the puncture side, and the circumference at 5 cm above the knee joint was 0.5 cm thicker than that before insertion, we recorded it as extremity swelling. Although there is no universal definition of phlebitis, we defined phlebitis as the presence of a linear red streak developing along the superficial veins from the catheter insertion site [6, 27]. When clinical signs of sepsis (e.g., fever, apnea, and tachycardia) were observed, a blood sample was drawn from a peripheral vein for blood culture, and if bacterial growth was detected in the blood culture, the ECC was removed, and the catheter tip was cultured. CRBSI was defined as the same bacterial colony cultured from peripheral blood and the ECC tip, signs of clinical deterioration, and absence of any other source of infection [27, 28]. Liquid or bloody exudation under the transparent dressing was documented as leakage.

### Data collection

In this study, all data were collected by certified ECC nurses and obtained from medical records, including general demographic information, ECC placement characteristics, tip position according to CXR, SR, IR, the difference between IR and SR, and catheter-related complications. All the authors had no access to information that could identify individual participants during or after data collection. Two certified ECC nurses independently analyzed ECG data. A radiologist and a certified ECC nurse independently reviewed CXR data, and a third radiologist was consulted for disagreements regarding CXR recordings. The follow-up time was from catheterization to catheter extraction.

### Data analysis

All data were collected in a software-based database for statistical analysis. Data analysis was performed using SPSS 21.0 (IBM Corp., New York, USA). Continuous data (e.g., gestational age, birth weight, and age at catheterization) are expressed as means with standard deviations, whereas categorical data (e.g., sex, insertion site, and insertion vein) are described as percentages. Between-group comparisons were performed using two-sided t-tests and χ^2^ tests for quantitative and qualitative variables, respectively. Binary logistic regression analysis was used to identify the potential risk factors of catheter-related complications. Our main analysis included only preterm infants with stable surface ECG and IC-ECG as well as interpretable CXR. A *P*-value < 0.05 was considered statistically significant.

## Results

### Patient characteristics

Between January 2019 and December 2021, a total of 303 preterm infants (321 ECCs) were enrolled in the present study, of whom 14 patients underwent single ECC replacement and two underwent double ECC replacement during the hospitalization period. Their gestational age ranged from 25 to 36.86 weeks, birth weight from 450 to 3430 g, catheterization age from 1 to 90 days, and ECC retention time from 1 to 40 days (Table 1).

**Table 1:** Patient demographic characteristics

Variable	Value
Gender, *n (%)*	
Male	151 (49.8)
Female	152 (50.2)
Gestational age, weeks	30.52 ± 2.62
Birth weight, g	1321.94 ± 471.64
Insertion site, *n (%)*	
Left	162 (50.5)
Right	159 (49.5)
Insertion vein, *n (%)*	
Femoral vein	216 (67.3)
Great saphenous vein	105 (32.7)
Age at catheterization, days	6.45 ± 13.56
ECC retention time, days	16.06 ± 7.54

### Effectiveness and quantitative changes of IC-ECG

The average reference length of the 321 ECCs was 14.05 ± 2.71 cm, and the actual insertion length was 12.83 ± 2.60 cm (t = 5.79, *P* < 0.001). All ECCs were confirmed to be in the IVC by CXR, of which 305 ECCs showed increased QRS amplitudes on their IC-ECGs. The matching rate between IC-ECG and CXR was 95.0%.

Subjects were stratified by whether the catheter was in the optimal central location (groups A and B). The catheters in group A were located in the optimal central location and did not need to be adjusted. The catheters in group B were located in a non-optimal central location and needed to be adjusted. ECG comparisons between the two groups are shown in Table 2. There were no differences in the demographic data between groups.

**Table 2:** ECG comparison between groups based on optimal (Group A) and non-optimal (Group B) catheter placement

Groups	N	IR (mv)	SR (mv)	IR-SR (mv)
Group A	220	1.69 ± 0.68	0.84 ± 0.51	0.85 ± 0.56
Group B	101	1.90 ± 0.67	0.88 ± 0.49	1.02 ± 0.65
t		-2.588	-0.618	-2.452
P		0.010*	0.537	0.015*

*Note*: IR, the amplitude of the IC-ECG QRS wave; SR, the amplitude of the surface ECG QRS wave; IR-SR, difference between IR and SR. *P < 0.05

### Safety of IC-ECG

All preterm infants experienced no adverse events during intraoperative IC-ECG. During postoperative catheter retention, catheter-related complications included extremity swelling (1.6%, n = 5), phlebitis (2.8%, n = 9), CRBSI (4.7%, n = 15), and leakage (1.6%, n = 5). The overall complication rate was 10.6% (n = 34), and the complication rates of the femoral and great saphenous veins were 5.0% (n = 16) and 5.6% (n = 18), respectively.

According to the occurrence of catheter-related complications, subjects were divided into case and control groups. There were significant differences in the ECC reference length, actual insertion length, catheter retention time, and insertion vein between the two groups (Table 3). Variables that were statistically significant (*P* < 0.05) as well as those that could have clinical meaning (the number of venipunctures and catheter adjustment) were included in the final logistic regression analysis. The results showed that the actual insertion length was a risk factor for catheter-related complications, and catheter retention time was a protective factor (Table 4).

**Table 3:** Univariate Comparison of Exposure Factors for Catheter-related Complications

Items	Case group N = 34	Control group N = 269(287ECCs)	t/χ2	*P*
Gender, *n (%)*			0.000	0.984
Male	17 (50.0)	134 (49.8)		
Female	17 (50.0)	135 (50.2)		
Gestational age, weeks	30.94 ± 2.43	30.46 ± 2.64	0.434	0.320
Birth weight, g	1374.74 ± 525.21	1315.26 ± 465.08	0.083	0.489
Age at catheterization, days	4.33 ± 7.63	6.70 ± 14.08	0.949	0.343
ECC reference length, cm	15.22 ± 2.58	13.91 ± 2.70	-2.699	0.007*
Actual insertion length, cm	13.99 ± 2.47	12.69 ± 2.59	-2.786	0.006*
Catheter retention time, days	13.29 ± 7.45	16.39 ± 7.50	2.275	0.024*
Number of venipunctures, time	1.03 ± 0.17	1.18 ± 0.59	1.457	0.146
Insertion site, *n (%)*			1.313	0.252
Left	14 (41.2)	148 (51.6)		
Right	20 (58.8)	139 (48.4)		
Insertion vein, *n (%)*			7.071	0.008*
Femoral vein	16 (47.1)	200(69.7)		
Great saphenous vein	18 (52.9)	87(30.3)		
Catheter adjustment, *n (%)*			0.809	0.369
No	21 (61.8)	199 (69.3)		
Yes	13 (38.2)	88 (30.7)		

*Note*: * *P* < 0.05

**Table 4:** Multivariate Analysis for Catheter-related Complications

Variable	OR (95% CI)	*P*
Actual insertion length, cm	1.222 (1.066–1.401)	0.004*
Catheter retention time, days	0.941 (0.890–0.995)	0.034*

*Note*: Values are presented as ORs with 95% CIs. Binary logistic regression analysis was performed. CI, confidence interval; OR, odds ratio. * *P* < 0.05

## Discussion

In recent years, IC-ECG has been widely used for locating the ECC tip, especially in neonates. Growing evidence suggests that IC-ECG is a safe, accurate, easy, and immediate approach for intraoperative ECC tip confirmation [6, 8, 10, 11, 14, 16–18, 20]. To our knowledge, this retrospective analysis is the first study to quantitatively describe the major changes in IC-ECG for ECC via the lower extremity and determine its effectiveness and safety, which extends upon previous findings.

Our study highlighted four findings. First, the reference length measured before insertion was higher than the actual insertion length guided by IC-ECG (*P* < 0.001). Second, a total of 321 ECCs were successfully placed in the IVC, of which 305 ECCs showed increased QRS amplitudes on their IC-ECGs (matching rate, 95.0%). Third, when ECCs were in the optimal central location, the IR (the QRS amplitude of IC-ECG) was 1.69 ± 0.68 mv, which was 0.85 ± 0.56 mv higher than the SR (the QRS amplitude of surface ECG). Finally, all preterm infants experienced no adverse events during intraoperative IC-ECG, and the overall complication rate for ECC was 10.6% during the maintenance period. In the logistic regression analysis, the actual insertion length was associated with an increased risk of complications and was considered an independent risk factor, while catheter retention time was a protective factor.

The ECC reference length is generally estimated by anatomical landmarks or through the formula method [25, 29–31]. In this study, the ECC reference length was based on anatomical markers, while the actual insertion length was determined by IC-ECG, and the difference between the two was statistically significant (*P* < 0.001). In neonates, increasing evidence has shown that IC-ECG-guided ECC placement can achieve more accurate tip positioning in the first attempt and reduce catheter-related complications [6, 8, 10, 14, 16, 17, 20, 32]. Thus, it is necessary and valuable to use IC-ECG to locate the catheter tip during ECC catheterization.

The effectiveness of IC-ECG in our study was 95.0%, which is consistent with that in previous reports (93.6–97.8%) [6, 8, 18]. In contrast with our findings, Zhou et al. [11] reported that the overall accuracy of IC-ECG-guided ECC placement was lower in neonates, with 74.7% (59/79) and 91.6% (33/36) in the upper and lower extremities, respectively. We speculated that there were several factors associated with this discrepancy. First, the study populations were different. Our study subjects were all premature infants with a lower weight, whereas in Zhou et al.’s study, both full-term and premature infants were included, and their findings showed that weight was associated with the accuracy of IC-ECG: the heavier the neonate, the lower the accuracy [11]. Second, the puncture sites of the two studies were different. In our study, all sites were in the lower extremity, while in Zhou et al.’s study, more sites were in the upper extremity. Finally, the IC-ECG equipment used in the two studies was different, which may influence the results and requires further study. However, Zhou et al. suggested that the accuracy of IC-ECG for ECC via the lower extremity was higher than that via the upper extremity.

The ECC tip position in the SVC region (head-neck or upper extremity) can be determined based on the dynamic morphological changes of the P wave on IC-ECG [5–17]. However, the IC-ECG characteristics for ECC located in the IVC (lower extremity) are different from those for ECC located in the SVC and have been sparsely reported. When the catheter tip is in the IVC, the IC-ECG is characterized first by an increase in QRS amplitude, followed by a larger P wave as the catheter enters the right atrium, which suggests that the catheter tip was too deep and should be withdrawn until the P wave reverts to normal [25, 33–35]. Consistent with our findings, three previous studies reported that the optimal position of the ECC tip via the lower extremity could also be determined by the gradually increasing QRS amplitude and P wave morphology [11, 18, 20]. Furthermore, our study quantified the magnitude of the increase in QRS amplitude (the QRS amplitude of IC-ECG was 0.85 ± 0.56 mv higher than that of surface ECG) when the catheter tip was optimally positioned. This finding filled the gap in previous research.

In this retrospective study, the puncture veins for lower extremity ECC included the femoral and great saphenous veins. Regardless of the vein selected, the catheter was inserted blindly. Catheter function and complications were evaluated daily, mainly according to the symptoms of catheter-related complications. The great saphenous vein, which is superficial, visible, and far from the perineal area, is the preferred vein for lower extremity ECC. However, our results show that the great saphenous vein has a higher complication rate. This may be because compared with the femoral vein, the saphenous vein has a long distance from the inserted site to the endpoint of the central position. This finding is also in good agreement with the previously reports that the larger contact area may lead to more frequent friction and thus a higher risk of phlebitis caused by mechanical stimulation [36]. In addition, in our study, most premature infants had edema, resulting in unclear greater saphenous veins, which increased the difficulty of puncture. Multiple punctures may have further increased the incidence of complications.

Our multivariate analysis indicated that the actual insertion length and catheter retention time were associated with catheter-related complications, with deeper insertion lengths and shorter retention times associated with lower safety. The actual insertion length determines the catheter tip location, which may have a significant impact on complications [37]. Previous studies have shown that noncentral ECC tip positioning was the only independent risk factor for nonselective ECC removal and was associated with an increased rate of catheter-related complications [38, 39]. Therefore, every effort should be made to achieve and maintain the central ECC tip location. Compared with the SVC, the IVC is longer, straighter, and less branched; hence, ECC insertion via the lower extremity can more easily be centrally located [11]. Multiple studies have shown that there is no significant difference in the overall complication rate between upper and lower extremity ECC [21, 22, 26, 31]. However, lower extremity ECC can significantly reduce the risk of malposition and catheter-related pleural effusion [18, 22]. In summary, we suggest that the lower extremity should be preferentially selected as the ECC insertion site. As for the retention time, one potential explanation is that catheter-related complications may lead to nonselective catheter removal, which may account for the reduced retention time [21, 22, 26, 32, 37].

There are several limitations to our study. First, this was a retrospective study conducted at a single center with a small sample size and could not determine the validity of IC-ECG method. Therefore, our study might provide limited generalizability for the application of IC-ECG guidance for ECC placement via the lower extremity in preterm infants. Second, we chose CXR to confirm the tip position, which is a relatively inaccurate, post-procedural, time-consuming, and harmful methodology, whereas real-time ultrasound is an accurate, intra-procedural, real-time, non-invasive, and safe methodology [15, 26]. Growing evidence suggests that real-time ultrasound is more accurate than conventional radiology because of its direct visualization of all venous districts and the catheter tip [26, 40, 41]. Unfortunately, real-time ultrasound was not available in our unit. Third, we only focused on the preterm infant population; thus, we cannot provide information on term infants and children. All our findings must be validated in future studies with a prospective, multi-center design, larger sample sizes, different populations, and more accurate confirmation method.

## Conclusions

In conclusion, this retrospective clinical study quantitatively demonstrated the major changes in IC-ECG for ECC insertion via the lower extremity in preterm infants. When the tips of ECCs were located in the optimal position, the QRS amplitude of IC-ECG was 0.85 ± 0.56 mv higher than that of surface ECG. Different from the upper extremity ECCs, the changes of QRS waves in lower extremity ECCs occurred earlier than the changes of P wave. Therefore, the method of sequentially observing the increase in QRS main wave amplitude combined with P wave morphology to locate the tip of lower extremity ECC is effective and safe. Our findings would facilitate the application of IC-ECG for ECC localization.

## Data Availability

All data generated or analyzed during this study are included in this published article.

## Abbreviations

ECC: Epicutaneo–cava catheter
IC: ECG–Intracardiac Electrocardiogram
ECG: Electrocardiogram
SVC: Superior Vena Cava
IVC: Inferior Vena Cava
IR: IC–ECG QRS amplitude
SR: Surface ECG QRS amplitude
CXR: Chest X–ray
